# Assessment of Ventricular-Arterial Coupling in Early Stage Middle-Aged Hypertensives

**DOI:** 10.21203/rs.3.rs-6189873/v1

**Published:** 2025-03-27

**Authors:** Andrea Vitali, Giuseppe Biondi Zoccai, George Booz, Raffaele Altara

**Affiliations:** UniCamillus University in Rome; National Institute of General Medical Sciences; National Institute of General Medical Sciences; Department of Anatomy & Embryology, Faculty of Health, Medicine and Life Sciences, Maastricht University, Maastricht, The Netherlands

**Keywords:** arterial elastance, arterial hypertension, pulse wave velocity, global longitudinal strain, myocardial work

## Abstract

**BACKGROUND.:**

Ventricular-arterial coupling (VAC) is altered by aging and cardiovascular comorbidities, indicating myocardial dysfunction and/or arterial stiffness. Our aim was to demonstrate whether lifestyle changes and anti-hypertensive drug treatment would improve VAC in recently diagnosed, early stage middle-aged hypertensives (HTN) without organ damage.

**METHODS.:**

Arterial elastance (Ea), carotid-femoral pulse wave velocity (cfPWV), global longitudinal strain (GLS), and myocardial work (MW) [global work index (GWI), global constructive work (GCW), global wasted work (GWW), and global work efficiency (GWE)] were investigated. This retrospective observational study involved 126 individuals (mean age 40 years; 55% female), divided into HTN and normotensives, NT. Clinical, echocardiographic and echo vascular parameters were assessed. Lifestyle changes were recommended for HTN. If blood pressure (BP) values still remained high, anti-hypertensive drug treatment was administered.

**RESULTS.:**

Higher values of systolic blood pressure (SBP), mean arterial pressure (MAP), heart rate (HR), GWI, GCW, and GWW were observed in HTN. By following lifestyle changes, BP [diastolic blood pressure (DBP) and MAP], HR, VAC, Ea, cfPWV, GWE, and GLS were changed in HTN; after 6 months of anti-hypertensive drug treatment, BP (SBP, DBP and MAP), HR, VAC, Ea, cfPWV, GWI, GCW, GWW, GWE, and GLS were found to be changed. VAC was linearly related to cfPWV and GLS at two follow ups. No statistically significant difference in VAC between HTN and NT was found.

**CONCLUSIONS.:**

Along with a decrease in BP, smoking cessation, and HR control highlighted a significant role in cardiovascular prevention by improvement of VAC, Ea, cfPWV, GLS and MW.

## Introduction

Ventricular-arterial coupling (VAC), defined as the ratio of two elastances, represents the relationship between the heart and the arterial system. This parameter (measured invasively or non-invasively) is altered by aging and following cardiovascular pathologies, indicating a deterioration of ventricular contractile function (myocardial dysfunction) and/or a stiffening of the arterial system (arterial stiffness). VAC can be calculated by various mathematical formulas, one of which is given by the ratio between the velocity of the arterial pressure wave (pulse wave velocity, PWV), estimated by various methods – including by vascular ultrasound through carotid-femoral pulse wave velocity (cfPWV) – and myocardial contractility, assessed through echocardiographic calculation of global ventricular deformation (global longitudinal strain, GLS).^1^

As myocardial work (MW) represents the force that the heart exerts, by means of the contraction of cardiomyocytes, its assessment offers an additional means to evaluate cardiac performance in relation to energy expenditure, influenced by arterial afterload (aortic pressure) and stroke volume (SV), across various clinical scenarios.^2^

This retrospective observational pilot-study was focused on middle-aged individuals recently diagnosed with early-stage hypertension [Stage I according to the 2024 European Society of Cardiology (ESC) guidelines] without organ damage (Suppl. Figures 1 & 2). Given that the parameters VAC, cfPWV, GLS, arterial elastance (Ea) and MW [in all its components: global work index (GWI), global constructive work (GCW), global wasted work (GWW), and global work efficiency (GWE)] have been primarily studied in cardiovascular diseases of aged populations, we sought to demonstrate whether these parameters were also altered in the early stages of arterial hypertension in middle-aged individuals. Additionally, we assessed whether normalization of blood pressure (BP) through lifestyle changes and/or anti-hypertensive drug treatment would lead to normalization of these parameters.^3–5^

## Material and Methods

### Study design.

This was a retrospective observational study. The medical records for three years (from 1 March 2021 to 1 March 2024) were screened in the Terni Health District and in other public health districts of the Umbria region (Italy). The study was approved by the Umbria Regional Ethics Committee (protocol nr. 4744/24) and by the Asl Umbria 2 (by executive decision nr. 0003982). The primary outcome was to evidence that lifestyle changes and antihypertensive therapy normalize the ultrasound parameter VAC in recently diagnosed, early stage hypertensives (HTN) without organ damage. The secondary outcomes were (a) demonstration that the non-invasively assessed ultrasound parameters - VAC, Ea, cfPWV, GLS, MW - show worse values in recently diagnosed early-stage HTN without organ damage compared to healthy individuals (normotensives, NT), and (b) demonstration that lifestyle changes and antihypertensive therapy normalize the parameters Ea, cf PWV, GLS, MW.

### Study protocol.

The study is divided in two parts. In the first part, recently diagnosed middle-aged early stage HTN, diagnosed within 3 months of the first cardiological visit without organ damage, were compared to NT. Selected patients were divided into two groups: group A (HTN) and group B (NT) at day 0 (zero). Both groups had the same range of age (30–49 years old), sexes and were enrolled by inclusion and exclusion criteria. In the second part, only HTN were investigated by the 2^nd^ and 3^rd^ cardiological visits (two 6 months follow-ups). Cardiological visits (case history and physical examination), general, clinical, electrocardiographic, echocardiographic and echo vascular parameters were assessed at day 0 and at the two follow-ups; laboratory data were assessed in this study only once, at day 0, as part of exclusion criteria (Suppl. Fig. 3).

### Participants:

A total of 672 medical records (493 pts excluded at 1st cardiological visit, 53 pts excluded between 1^st^ and 2^nd^ cardiological visit) were analyzed, and 126 patients selected according to the selection criteria of the study. HTN were defined as clinic systolic blood pressure (SBP) range 140–159 mmHg; diastolic blood pressure (DBP) range 90–99 mmHg. NT [normal or elevated blood pressure (BP) individuals, but not hypertensive individuals] were defined as normal clinic BP (< 140 mmHg SBP and < 90 mmHg DBP).

### inclusion criteria:

30–49 years old pts of both sexes (HTN and NT); HTN selected had high BP at 1^st^ cardiological visit or within three months from the 1^st^ cardiological visit (assessed at the patient’s home or by family or occupational physician); HTN selected had to be included in the I stage (early stage) hypertension: SBP range 140–159 mmHg; DBP range 90–99 mmHg.

### Exclusion criteria:

Individuals with hypertension over three months by the 1st cardiological visit; Individuals with a remote history of hypertension; SBP > 160 mmHg and/or DBP > 100 mmHg; HTN with evidence of increased BP during the study (BP assessed during the follow-up cardiological visit or at home > 160/100 mmHg); Altered laboratory parameters: Hemoglobin (Hb) < 12 g/dl (female) < 14 g/dl (male) or > 16 g/dl (female) > 18 g/dl (male); White Blood Cells (WBC) < 4000/mm^3^ or > 10000/mm^3^; Platelets (PLT) < 150000/mm^3^ or > 400000/mm^3^; creatinine > 1 mg/dl; Glomerular Filtration Rate (GFR) < 60 ml/min/1.73 mq, albuminuria > 30 mg/g; natremia < 130 mEq/l or > 146 mEq/l; kalemia < 3,3 mEq/l or > 5,2 mEq/l; glycemia < 60 mg/dl or > 100 mg/dl; total cholesterol > 200 mg/dl; trygliceridemia > 150 mg/dl; low-density lipoproteins (LDL) > 115 mg/dl; high-density lipoproteins (HDL) < 35 mg/dl; Altered electrocardiographic parameters: no sinus rhythm; electrocardiographic criteria for left ventricular (LV) hypertrophy; Altered echocardiographic parameters: LV mass index > 115 g/m^2^ (male) > 95 g/m^2^ (female), interventricular septum thickness (IVST) > 11 mm, posterior wall thickness (PWT) > 11 mm, relative wall thickness (RWT) > 0,43, LV end diastolic diameter (LVEDD) > 60 mm, LV end systolic diameter (LVESD) > 45 mm, LV ejection fraction (EF) < 50%, Left Atrial Volume index (LAVi) > 34 ml/mq, E wave/e’ wave ratio (E/e’) > 14; valvular pathologies (moderate or severe stenosis and/or insufficiency); altered echo vascular parameters: carotid Intima-Media Thickness (IMT) > 0,9; presence of carotid plaques; Ankle Brachial Index (ABI) < 0,9 or > 1,4; Individuals with hypertension or cardiovascular organ damage.

### Interventions/Procedures:

HTN were recommended lifestyle changes and anti-hypertensive medication (medication recommended if BP not normalized). No invasive interventions or procedures on patients was performed.

### Measurements:

In the assessment of the medical records, the following parameters were collected. General and clinical parameters: Age, Gender, SBP, DBP, Mean Arterial Pressure (MAP), Body Surface Area (BSA), current smoking, current drinking; Laboratory parameters: Complete Blood Count (CBC), creatinine, GFR, albuminuria, natremia, kalemia, glycemia, complete lipid profile: total cholesterol, triglyceridemia, LDL, HDL; Electrocardiographic parameters: sinusal rhythm, heart rate (HR), electrographic signs of LV hypertrophy; Echocardiographic parameters: LV mass index, IVST, PWT, RWT, LVEDD, LVESD, EF, SV, Stroke volume index (Svi), LAVi, E/e’, MW (GWI, GCW, GWW, GWE), GLS; Echo vascular parameters: carotid IMT, presence of carotid plaques, cfPWV, ABI. BP was determined by brachial BP measurements reported in the medical record. HR was calculated by electrocardiogram. BSA was calculated by the Mosteller equation. VAC and Ea were calculated as VAC = cfPWV / GLS and Ea = (SBP × 0,9) / SV, respectively.

### Assessment of BP.

In all cardiological visits the patients were asked to relax for 15 minutes in a quiet room before measuring their BP (office BP), after which their weight and height were measured in order to calculate the BSA. SBP and DBP were measured three times using a sphygmomanometer and the average of the three measurements was used.

#### Day zero:

On day zero, the first cardiological visit assessed that 179 people were considered valid for inclusion. Between the 1^st^ and the 2^nd^ visits 53 pts were excluded. Of these 126 selected individuals, 68 were included in the group A (HTN), 58 in the group B (NT). In both groups, smokers and alcohol binge individuals (drinkers) were identified: group A 68 HTN participants, 38/68 smokers, 12/68 drinkers; group B 58 NT participants, 1/58 smokers, 0/58 drinkers.

#### 6 and 12 months follow up:

Six months lifestyle changes were recommended to HTN (stop smoking, stop alcohol intake, reduce salt intake, foster weight loss, 30 minutes five times a week of moderate intensity aerobic exercise). After a period of 6 months, in HTN group, if SBP kept on being in the range 140–159 mmHg and DBP in the range 90–99 mmHg, antihypertensive drug therapy was administered (monotherapy or dual). In this study, the antihypertensive drug administered was ACE inhibitor Perindopril (at a dosage of 5 mg/day). The dual drug therapy administered was Perindopril/Amlodipine, at a dosage of 5 mg/5 mg per day. In the first follow-up at the 2^nd^ cardiological visit (6 months after day zero), after lifestyle changes, of the 68 HTN, 12/68 had a normalization of BP; 56/68 HTN were required to start drug antihypertensive treatment: 29 out of 56 HTN in monotherapy; 27 out of 56 HTN in dual therapy. 53 HTN were excluded in the 1^st^ follow-up at the 2nd cardiological visit.

After the first cardiological visit one patient stopped smoking and one patient stopped habitual use of alcohol. In the second follow-up at the 3^rd^ cardiological visit (12 months after day zero) it has been assessed that the 12 HTN with normalization of BP at the first follow-up maintained normal BP, the 56 HTN treated with medication continued to take antihypertensive therapy and no HTN were excluded at the 2^nd^ cardiological follow-up. After the 2^nd^ cardiological visit an additional two patients stopped smoking and two patients stopped habitual use of alcohol.

### Statistical analysis:

Statistical analysis was performed using Graphpad Prism Ver. 10.3.0 (GraphPad Software, San Diego, California, USA). In the first part of the study continuous variables with a normal distribution were expressed as mean ± SEM and HTN compared to NT using 2-way ANOVA. In the second part, 1-way ANOVA was used and multiple linear regression was performed to assess the impact of each variable on VAC. A value of p < 0.05 was considered statistically significant.

## Results

**Figure 1:**
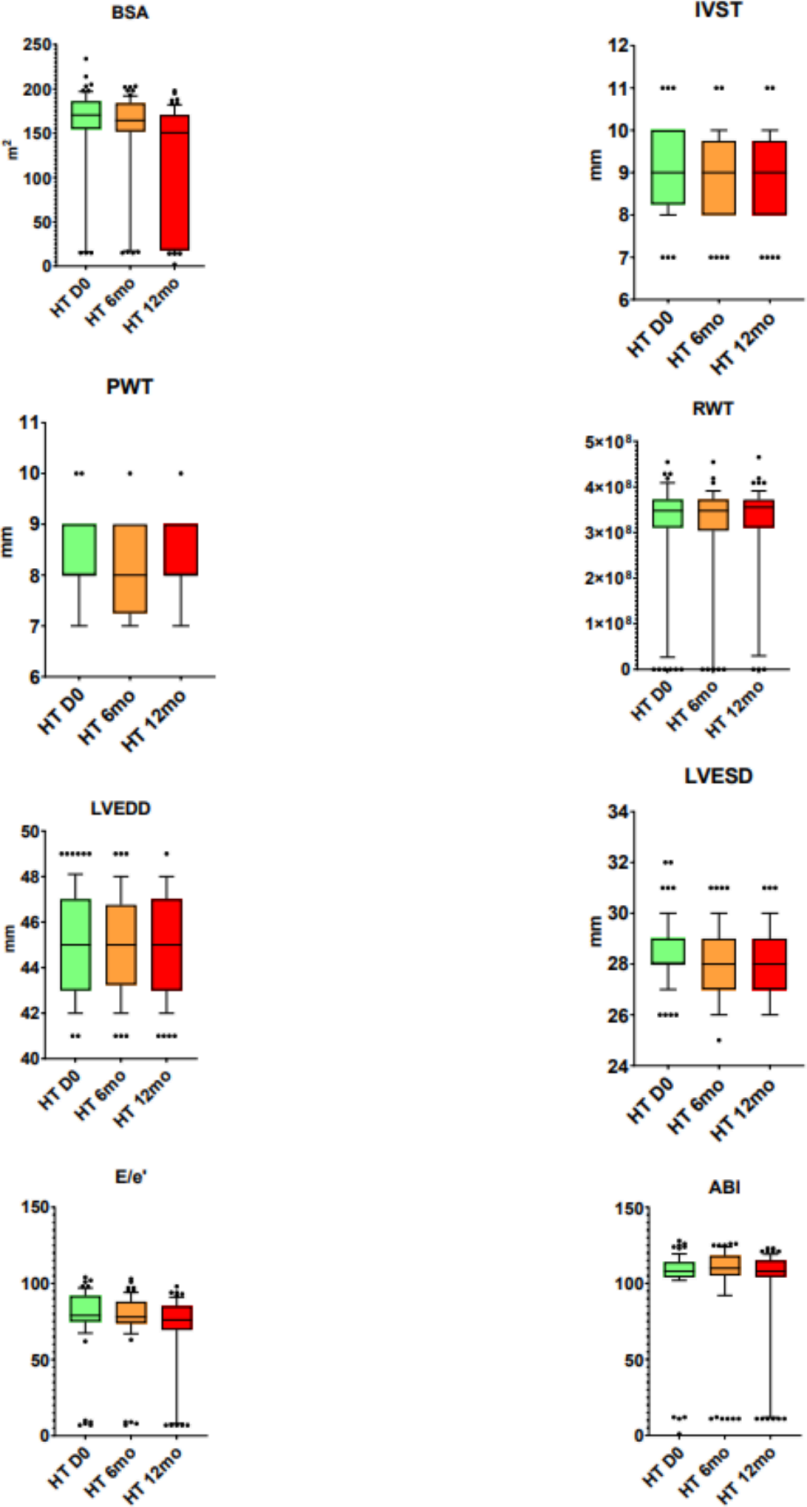
Clinical and echocardiographic parameters in HTN. BSA, body surface area; IVST, interventricular septum thickness; PWT, posterior wall thickness; RWT, relative wall thickness; LVEDD, left ventricular end diastolic diameter; LVESD, left ventricular end systolic diameter; E/e’ ratio, E wave / e’ wave ratio; ABI, ankle brachial index.

**Figure 2:**
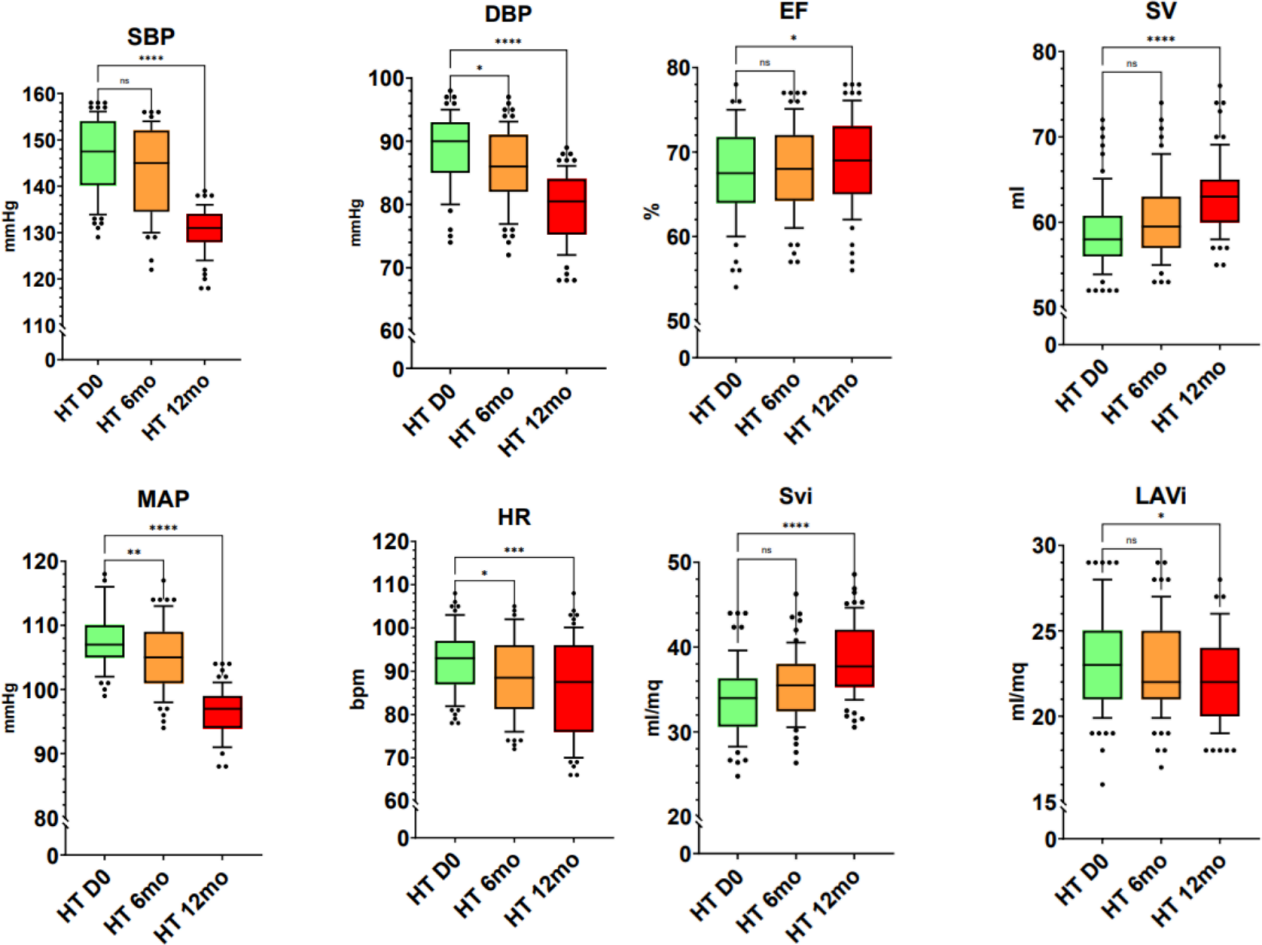
BP, HR and echocardiographic parameters in HTN. SBP, systolic blood pressure; DBP, diastolic blood pressure; MAP, mean arterial pressure; HR, heart rate; EF, ejection fraction; SV, stroke volume; Svi, stroke volume index; LAVi, left atrial volume index.

**Figure 3:**
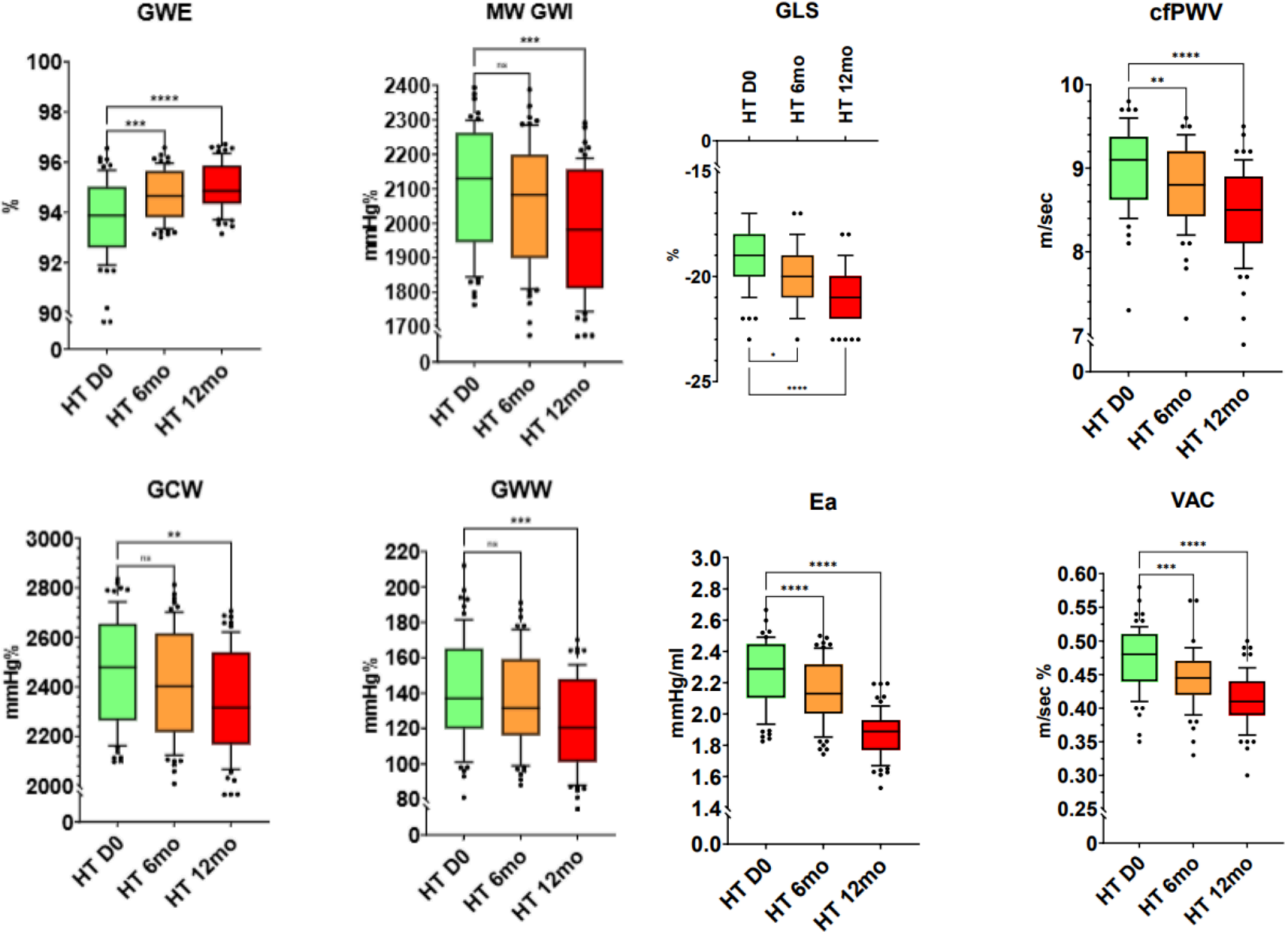
MW components, echocardiographic and echo vascular parameters GLS, cfPWV, Ea, VAC in the study population (HTN). MW, Myocardial Work; GWI, global work index; GCW, global constructive work; GWW, global wasted work; GWE. global work efficiency; GLS, global longitudinal strain; cfPWV, carotid femoral Pulse Wave Velocity; Ea, arterial elastance; VAC, ventricular-arterial coupling.

Out of 672 medical records, 126 individuals (aged 30–49 years old) were selected and included in this retrospective observational pilot-study (Table 1). They were divided in two groups: group A (HTN) and group B (NT). In HTN compared to NT, an increase in SBP, MAP, HR, MW GWI, MW GCW, and MW GWW was observed. There was a trend of increased values for DBP (Table 2). In HTN, at day 0 (zero) and after the two follow-ups, the results of clinical and echographic parameters were as follows (Fig. 1, 2, 3). A decrease was observed in the values of SBP at 12 months, DBP at 6 and 12 months, MAP at 6 and 12 months, and HR at 6 and 12 months. Despite the positive trend towards improvement at 6 months for SBP, this did not reach a significant reduction. In HTN, the echocardiographic parameters identified an increase in EF and SV/Svi at 12 months and a reduction in LAVi at 12 months (Fig. 2).

In HTN, a reduction in GWI, GCW, and GWW in the investigated MW components at 12 months and an improvement in GWE at 6 and 12 months were observed. Despite the trend at 6 months of a reduction in GWI, GCW, and GWW, none reached statistical significance. In HTN, a reduction in GLS, cfPWV, Ea, and VAC was observed at 6 and 12 months (Fig. 3).

**Figure 4:**
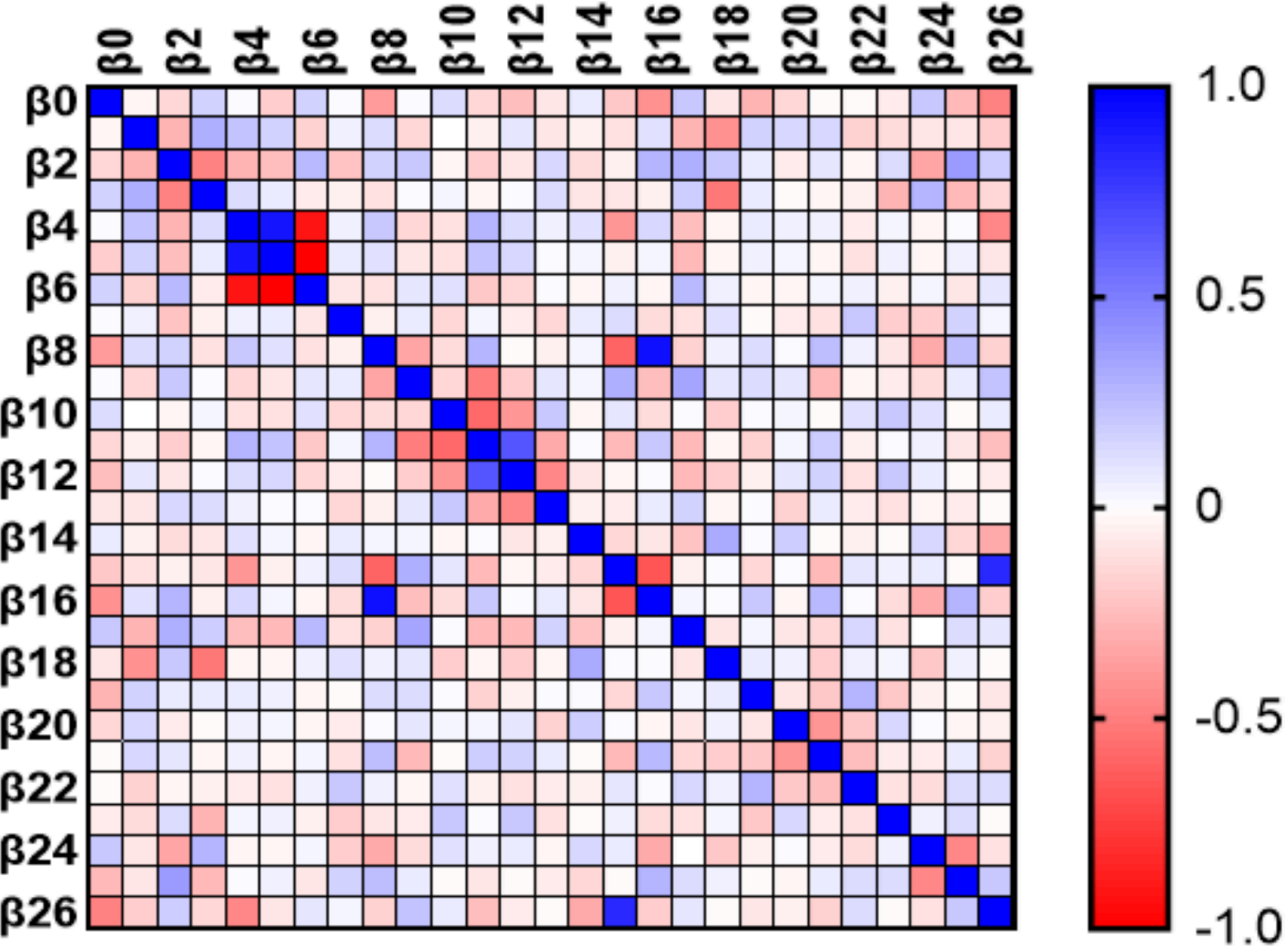
Multiple linear regression (impact of each variable on VAC) HTN at Day 0. B0, intercept; B1, age; B2, current smokers; B3, current drinkers; B4, systolic blood pressure; B5, diastolic blood pressure; B6, mean arterial pressure; B7, heart rate; B8, body surface area; B9, interventricular septum thickness; B10, posterior wall thickness; B11, relative wall thickness; B12, left ventricular end diastolic diameter; B13, left ventricular end systolic diameter; B14, ejection fraction; B15, stroke volume; B16, stroke volume index; B17, left atrial volume index; B18, E/e’ ratio; B19, global work efficiency; B20. global work index; B21, global constructed work; B22, global wasted work; B23, global longitudinal strain; B24, carotid femoral Pulse Wave Velocity; B25, ankle-brachial index; B26, arterial elastance.

**Figure 5:**
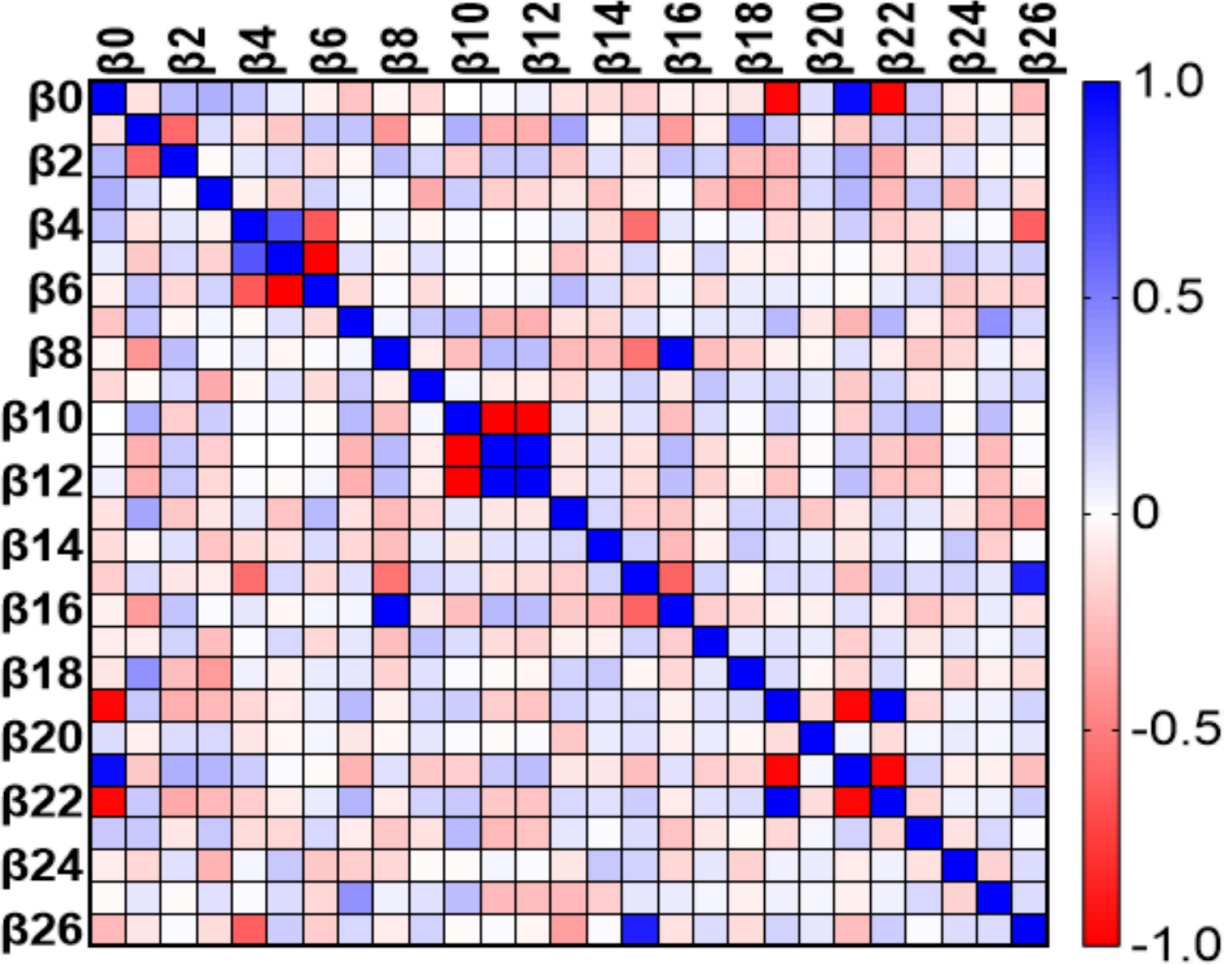
Multiple linear regression (impact of each variable on VAC) HTN at 6 months. B0, intercept; B1, current smokers; B2, current drinkers; B3, age; B4, systolic blood pressure; B5, diastolic blood pressure; B6, mean arterial pressure; B7, heart rate; B8, body surface area; B9, interventricular septum thickness; B10, posterior wall thickness; B11, relative wall thickness; B12, left ventricular end diastolic diameter; B13, left ventricular end systolic diameter; B14, ejection fraction; B15, stroke volume; B16, stroke volume index; B17, left atrial volume index; B18, E/e’ ratio; B19, global work efficiency; B20, global work index; B21, global constructed work; B22, global wasted work; B23, global longitudinal strain; B24, carotid femoral Pulse Wave Velocity; B25, ankle-brachial index; B26, arterial elastance.

**Figure 6:**
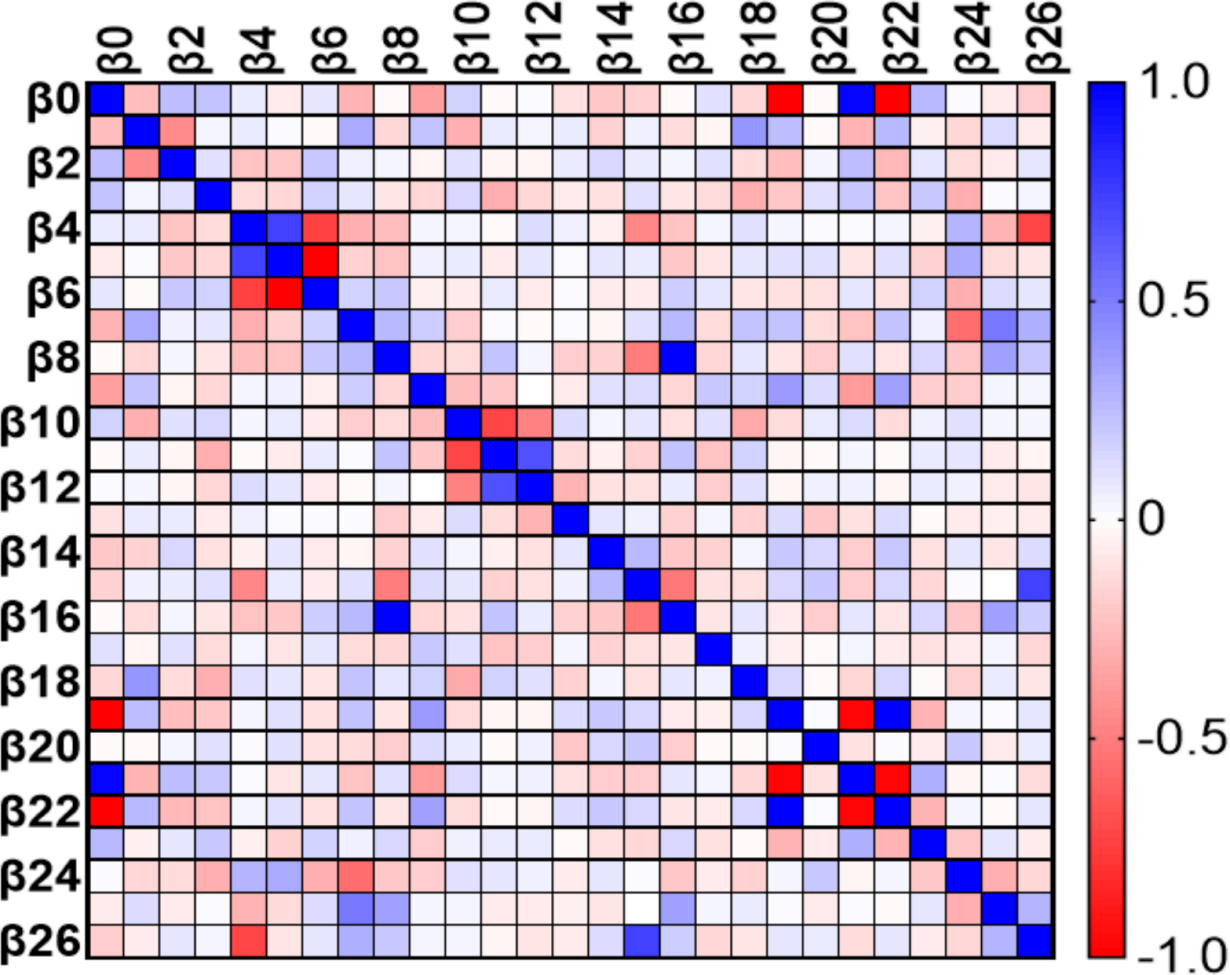
Multiple linear regression (impact of each variable on VAC) HTN at 12 months. B0, intercept; B1, current smokers; B2, current drinkers; B3, age; B4, systolic blood pressure; B5, diastolic blood pressure; B6, mean arterial pressure; B7, heart rate; B8, body surface area; B9, interventricular septum thickness; B10, posterior wall thickness; B11, relative wall thickness; B12, left ventricular end diastolic diameter; B13, left ventricular end systolic diameter; B14, ejection fraction; B15, stroke volume; B16, stroke volume index; B17, left atrial volume index; B18, E/e’ ratio; B19, global work efficiency; B20, global work index; B21, global constructed work; B22, global wasted work; B23, global longitudinal strain; B24, carotid femoral Pulse Wave Velocity; B25, ankle-brachial index; B26, arterial elastance.

At day 0, the variables MW GWI, MW GWW, current drinkers, and E/e’ were linearly related to VAC (Fig. 4). At 6 months the variables BSA, PWT, RWT, LVEDD, SVi, GLS, and cfPWV were found to be linearly related to VAC (Fig. 5). At 12 months the variables GLS, and cfPWV were linearly related to VAC (Fig. 6). Analysis indicated a strong trend to linear correlation to VAC for BSA, SBP, DBP, MAP, and Ea, although none were statistically significant.

## Discussion

In our study, we found no difference in VAC between HTN and NT groups. Along with HR and BP specifically SBP and MAP (with a positive trend towards significance for DBP), MW (in its components GWI, GCW, and GWW) was found to be higher in HT than NT. By assessment of the HTN group at the two follow-ups at 6 and 12 months, BP, EF, MW (in its components GWI, GCW, and GWW), VAC, Ea, cfPWV and GLS were significantly improved. Analysis of the impact of VAC on the parameters investigated in this study highlighted that VAC was linearly related to cfPWV and GLS after lifestyle changes and hypertensive drug treatment. In addition to cfPWV and GLS, MW (in its components GWI and GWW), current drinking and E/e’ were found to be linearly related to VAC at day 0, whereas the variables BSA, PWT, RWT, LVEDD, and SVi, were found to be linearly related to VAC at 6 months.

Notwithstanding no significant difference in VAC, significant difference in BP, HR and MW was observed between the two groups, highlighting the pre-clinical role of an increase in sympathetic activity in the onset of hypertension even in middle-aged HTN. Furthermore, along with EF and GLS, MW components were valid indicators of LV systolic function, even at the early onset of hypertension, providing a more comprehensive appraisal of LV systolic function.^6,7,8^

Despite the positive trend of lifestyle changes at 6 months, the improvement of the parameters VAC, Ea, MW, cfPWV, EF, and GLS was more evident after the onset of antihypertensive therapy, demonstrating the benefit of a further reduction in BP. These data align with the current guidelines on further restricting BP in terms of normal ranges, highlighting the paramount impact of BP in the management of sub-clinical hypertension.^9^

As a consequence of the improvement of VAC, Ea, MW, cfPWV, EF, and GLS by lowering of BP and HR and changes in lifestyle (such as body weight loss, physical exercise, smoking cessation and reduction in salt and alcohol consumption), the improvement in systolic function by reduction of sympathetic overdrive and its significant effect on BP and HR was evidenced. In addition to aging as an independent contributor to the deterioration of diastolic function, the significant correlation between the echocardiographic parameter E/e’ ratio and VAC in HT was observed by echocardiographic signs of an early effect of high BP values on diastolic function.^10,11^

Despite no statistically significance, the increase in VAC in the young hypertensive population compared to the healthy individuals was based on an increase in cfPWV and a reduction in GLS. The two determinants cfPWV and GLS for the calculation of VAC were found significantly related to VAC at 6 and 12 months, easily predictable as VAC = PWV / GLS, deducing that simultaneously arterial stiffness and LV systolic function are progressively altered.^12,13^ This is dependant upon the progressive hardening of the arteries by aging (mostly in the population with exposure to risk factors such as tachycardia, high BP and smoking) that determined a progressive increase in the pulse wave transmission with consequent effect given by the return to the heart of the anterograde pulse wave at an earlier time point during the late systolic phase of the cardiac cycle. As a consequence of this, constantly increased afterload affected a progressive impairment of cardiac function with progressive reduction of GLS.^14^ Concerning the effect of medical treatment on BP, BP control occurred with antihypertensive drugs in monotherapy and dual therapy. As predictable, dual drug antihypertensive treatment was more effective in reducing both BP and VAC compared to single drug antihypertensive treatment.^3,9^

As demonstrated by other research, VAC was associated with the prevalence of both hypertension and other cardiovascular risk factors, such as active smoking, alcohol consumption, and increased HR, highlighting reports that it is the most significant predictor of risk.^1,15,16^ In agreement with the studies of Holm H et al. and Stoichescu-Hogea G et al., VAC and cfPWV were found higher and GLS lower than in the control population. The former was found to be especially associated with age and active smoking; the latter found to be worse in coronary artery disease and HTN vs HTN vs group control. Compared to the aformentioned studies, the investigated population in our study did not have additional cardiovascular risk factors or organ damage, highligthing that in early stage of hypertension these parameters were not statistically significant.^2,12,13,15,17,18^ The finding of no significant difference in VAC was mainly due to the small sample size and lack of longitudinal analysis in NT. Furthermore, as the investigated population was not elderly and without cardiovascular diseases, it was demonstrated that this parameter was not altered in middle-aged HTN at early stage of hypertension. As left VAC represents both a parameter that characterizes the energetic cost, particularly when the LV function is changed, and a hemodynamic measure linked to patient outcomes,^19,20^ this highlights the importance of observing small changes in the pre-clinical context, such as arterial hypertension without organ damage.^1^ On the other hand, in the population studied, MW was demonstrated to be sensitive to impairment even in young HTN, highlighting it to be more affected at early stages from augmentations in sympathetic increases in BP and HR. In addition, as MW was observed to be statistically higher in HT without other cardiovascular risk factors or organ damage, this parameter can account for BP and myocardial energy consumption at the onset of hypertension and not only in overt cardiovascular diseases.^2,10,21^

The NORRE substudy, was the first to offer data on age-related changes in GWI and GCW among both males and females. In women, values increased, whereas in men, they remained unchanged, with an increase primarily associated with elevated SBP alone. Additionally, GWW and GWE values were found to increase in men and decrease in women, with no significant age-related differences (Table 6).^22^ GWI represents the area within the LV loop quantifying the work performed by the LV throughout the entire mechanical energy between MVC and MVO; GCW represents the positive work performed by shortening during IVC and systole and the negative work by lengthening during filling, quantifying the energy consumed by the myocardium that effectively contributes to CO; GWW represents negative work by lengthening during IVC and systole and positive work by shortening during IVR, quantifying the wasted energy by the myocardium that does not contribute to CO. As a consequence of this, to complete the mechano-energetic study, the assessment of MW adds, through its components (GWI, GCW, GWW, GWE), a greater definition of myocardial energy expenditure both in the evaluation of the amount of MW performed by the left ventricle during systole and the amount of MW that participates or does not in ejection. We observed that at early stage of arterial hypertension, GWI, GCW, GWW are higher indicating an increase in MW expenditure. Through lifestyle changes and anti-hypertensive drug treatment, these parameters tend to be renormalized, indicating a better mechano-energetic efficiency.^2^ Therefore, myocardial mechano-energetic efficiency represented as a surrogate of MW was found to be more sensitive and with higher impact than VAC and other parameters studied at early stage of hypertension in middle-aged HTN.^18,21,23^

Used with permission from Dr. Federica Ilardi, April 8, 2024.

In light of these results, in addition to BP and HR controls, the sub-clinical assessment of LV systolic and diastolic functions can be investigated in clinical practice in young people at early stage of hypertension by calculating MW and the E/e’ ratio, respectively. Longitudinal studies with larger sample of young HT could provide additional robust results in order to define the significant role of VAC and the other parameters investigated in this study in terms of cardiovascular prevention for more accurate management and assessment in clinical practice.

### Limitations of the study

This study has several limitations. The number of people investigated was small and the timeframe brief. A longitudinal study on the healthy population was not performed, so that the HTN and NT groups were not compared at 6 and 12 months. Also, VAC was non-invasively calculated. This study did not analyze all stages of hypertension and HT with organ damage.

## Conclusions

VAC was found to be advantageous in early stage middle-aged HT for the assessment of the cardiovascular system. In addition to VAC, the parameters Ea, MW, cfPWV, GLS, and EF were highlighted to be altered even in early stage of hypertension and statistically improved after lifestyle changes or antihypertensive drug treatment. Since hypertension is both an important independent risk factor for cardiovascular disease and endothelial dysfunction, this study highlights the clinical impact of cardiovascular prevention in young HT, particularly in active smokers and drinkers with increased HR. Despite VAC being easily determined at the patient’s bedside, it still remains neglected. Further studies are needed to demonstrate the effective introduction of this parameter into clinical practice.

## Figures and Tables

**Table 1:** HTN vs NT, current smokers and drinkers.

	NT D0	HT D0
Patients	58 (25 M; 33 F)	68 (32 M; 36 F)
Current smokers	1	38
Current drinkers	0	12

NT D0, normotensives at day 0; HT D0, hypertensives at day 0; M, male; F, female.

**Table 2:** HTN vs NT. Mean ± SEM.

	NT D0	HT D0	
	Mean ± SEM	Mean ± SEM	p-value
AGE	40.21 ±0.87	38.16 ± 0.7	0.81
SBP	124.29 ±1.12	146.65 ± 1.00	**0.01**
DBP	71.79 ± 0.92	88.59 ± 0.69	0.05
MAP	89.26 ± 0.86	107.94 ± 0.54	**0.03**
HR	70.48 ± 1.40	92.24 ± 0.90	**0.01**
BSA	1.83 ± 0.03	1.76 ± 0.02	> 0.99
IVST	7.97 ± 0.08	9.04 ± 0.11	0.9
PWT	7.48 ± 0.07	8.18 ± 0.09	0.94
RWT	0.35 ± 0.00	0.36 ± 0.00	> 0.99
LVEDD	43.88 ± 0.29	45.16 ± 0.27	0.88
LVESD	27.33 ± 0.12	28.37 ± 0.16	0.9
EF	63.16 ± 0.76	67.09 ± 0.67	0.65
SV	62.67 ± 0.76	58.81 ± 0.56	0.66
Svi	34.91 ± 0.71	33.94 ± 0.51	0.91
LAVi	24.43 ± 0.40	23.38 ± 0.37	0.9
E/e’	7.02 ± 0.11	8.31 ± 0.12	0.88
MW GWE	0.95 ± 0.00	0.94 ± 0.00	> 0.99
MW GWI	1727.03 ± 16.56	2092.29 ± 21.28	**< 0.001**
MW GCW	2049.79 ± 18.06	2460.19 ± 26.47	**< 0.001**
MW GWW	78.88 ± 1.38	141.46 ± 3.60	**< 0.001**
Strain GLS	20.64 ± 0.21	19.28 ± 0.17	0.88
cfPWV	7.39 ± 0.12	9.01 ± 0.06	0.85
ABI	1.08 ± 0.01	1.10 ± 0.01	> 0.99
Ea (SBP × 0.9 / SV)	1.80 ± 0.03	2.26 ± 0.03	0.96
VAC (PWV/GLS)	0.36 ± 0.01	0.44 ± 0.01	0.99

NT D0, normotensives at day zero; HT D0, hypertensives at day zero; SBP, systolic blood pressure; DBP, diastolic blood pressure; MAP, mean arterial pressure; HR, heart rate; BSA, body surface area; IVST, interventricular septum thickness; PWT, posterior wall thickness; RWT, ratio of wall thickness; LVEDD, left ventricular end diastolic diameter; LVESD, left ventricular end systolic diameter; EF, ejection fraction; SV, stroke volume; SVI, stroke volume index; LAVi, left atrial volume index; MW, Myocardial Work; GWI, global work index; GCW, global constructive work; GWW, global wasted work; GWE, global work efficiency; GLS, global longitudinal strain; cfPWV, carotid femoral Pulse Wave Velocity; ABI, ankle-brachial index; Ea, arterial elastance; VAC, ventricular-arterial coupling.

**Table 3:** Analysis by multiple linear regression to the variable VAC at Day 0.

	HT0		
	R^2^	P value	95% CI (asymptotic)
Intercept		**0.0388**	0.2775 to 10.01
Current smokers	0.6632	0.2019	−0.2593 to 0.05649
Current drinkers	0.6149	**0.0042**	0.09662 to 0.4813
AGE	0.7979	0.9394	−0.01708 to 0.01843
SBP	0.9945	0.7591	−0.08686 to 0.06382
DBP	0.9965	0.8597	−0.1482 to 0.1242
MAP	0.9973	0.8663	−0.2154 to 0.1821
HR	0.3293	0.3894	−0.004299 to 0.01081
BSA	0.9689	0.0809	−2.748 to 0.1660
IVST	0.7915	0.9773	−0.1125to 0.1094
PWT	0.8171	0.3853	−0.08061 to 0.2045
RWT	0.9139	0.5034	−5.450 to 2.719
LVEDD	0.8286	0.9356	−0.05211 to 0.04808
LVESD	0.6088	0.6932	−0.04557 to 0.06789
EF	0.4853	0.2165	−0.01863 to 0.004349
SV	0.9874	0.099	−0.01466 to 0.1638
SVi	0.9797	0.0723	−0.1465 to 0.006629
LAVi	0.7278	0.1957	−0.01007 to 0.04769
E/e’	0.7396	**0.0038**	−0.2287 to −0.04723
MW GWE	0.5573	0.4632	−2.158 to 1.000
MW GWI	0.531	**0.0017**	−0.001015 to −0.0002518
MW GCW	0.5605	0.0786	−3.383e-005 to 0.0005998
MW GWW	0.7021	**0.0323**	0.0002753 to 0.005934
Strain GLS	0.4143	0.9235	−0.04511 to 0.04099
cfPWV	0.6977	0.0706	−0.01392 to 0.3313
ABI	0.5125	0.1954	−1.309 to 0.2759
Ea (SBP × 0.9 / SV)	0.9876	0.2447	−0.7776 to 2.965

HT D0, hypertensives at day zero; SBP, systolic blood pressure; DBP, diastolic blood pressure; MAP, mean arterial pressure; HR, heart rate; BSA, body surface area; IVST, interventricular septum thickness; PWT, posterior wall thickness; RWT, ratio of wall thickness; LVEDD, left ventricular end diastolic diameter; LVESD, left ventricular end systolic diameter; EF, ejection fraction; SV, stroke volume; SVI, stroke volume index; LAVi, left atrial volume index; MW, Myocardial Work; GWI, global work index; GCW, global constructive work; GWW, global wasted work; GWE, global work efficiency; GLS, global longitudinal strain; cfPWV, carotid femoral Pulse Wave Velocity; ABI, ankle-brachial index; Ea, arterial elastance; VAC, ventricular-arterial coupling; CI, confidence interval.

**Table 4:** Analysis by multiple linear regression to the variable VAC at 6 months.

	HT6mo		
	R^2^	P value	95% CI (asymptotic)
Intercept		0.9223	−1.320 to 1.198
Current smokers	0.6514	0.648	−0.002207 to 0.003509
Current drinkers	0.5768	0.2352	−0.005395 to 0.001363
AGE	0.787	0.3579	−0.0001719 to 0.0004653
SBP	0.9965	0.2033	−0.0005639 to 0.002570
DBP	0.9971	0.1467	−0.0006938 to 0.004493
MAP	0.9982	0.1311	−0.006781 to 0.0009127
HR	0.4636	0.2288	−5.061e-005 to 0.0002056
BSA	0.9917	**0.0418**	0.002353 to 0.1185
IVST	0.6724	0.9519	−0.001646 to 0.001550
PWT	0.9981	**0.0039**	0.01321 to 0.06463
RWT	0.9986	**0.0053**	−1.460 to −0.2718
LVEDD	0.9924	**0.0078**	−0.01132 to −0.001828
LVESD	0.6765	0.7537	−0.0008960 to 0.001228
EF	0.4162	0.573	−0.0002677 to 0.0001502
SV	0.9971	0.3388	−0.004876 to 0.001717
SVi	0.9943	**0.0493**	9.617e-006 to 0.005613
LAVi	0.6189	0.5069	−0.0006361 to 0.0003193
E/e’	0.6216	0.593	−0.001873 to 0.001084
MW GWE	0.9957	0.2755	−0.5962 to 2.038
MW GWI	0.4854	0.0621	−1.287e-005 to 3.328e-007
MW GCW	0.9818	0.5162	−3.804e-005 to 1.941e-005
MW GWW	0.9963	0.2384	−0.0002039 to 0.0007965
Strain GLS	0.3557	**< 0.0001**	−0.02262 to −0.02113
cfPWV	0.6052	**< 0.0001**	0.04522 to 0.05089
ABI	0.4202	0.0725	−0.001392 to 0.03048
Ea (SBP × 0.9 / SV)	0.9972	0.9529	−0.08216 to 0.07747

HT 6mo, hypertensives at 6 months; SBP, systolic blood pressure; DBP, diastolic blood pressure; MAP, mean arterial pressure; HR, heart rate; BSA, body surface area; IVST, interventricular septum thickness; PWT, posterior wall thickness; RWT, ratio of wall thickness; LVEDD, left ventricular end diastolic diameter; LVESD, left ventricular end systolic diameter; EF, ejection fraction; SV, stroke volume; SVI, stroke volume index; LAVi, left atrial volume index; MW, Myocardial Work; GWI, global work index; GCW, global constructive work; GWW, global wasted work; GWE, global work efficiency; GLS, global longitudinal strain; cfPWV, carotid femoral Pulse Wave Velocity; ABI, ankle-brachial index; Ea, arterial elastance; VAC, ventricular-arterial coupling; CI, confidence interval.

**Table 5:** Analysis by multiple linear regression to the variable VAC at 12 months.

	HT12mo		
	R^2^	P value	95% CI (asymptotic)
Intercept		0.7223	−1.475 to 1.031
Current smokers	0.5734	0.8328	−0.002490 to 0.002016
Current drinkers	0.4336	0.8408	−0.002802 to 0.002292
AGE	0.697	0.8102	−0.0002050 to 0.0002608
SBP	0.99	0.4023	−0.002247 to 0.0009198
DBP	0.996	0.5154	−0.002881 to 0.001468
MAP	0.9963	0.4945	−0.002157 to 0.004393
HR	0.6715	0.5565	−7.849e-005 to 0.0001437
BSA	0.9943	0.9639	−0.06254 to 0.06542
IVST	0.685	0.7643	−0.001623 to 0.001201
PWT	0.8296	0.415	−0.001407 to 0.003344
RWT	0.8901	0.679	−0.06818 to 0.04485
LVEDD	0.7574	0.788	−0.0008299 to 0.0006337
LVESD	0.4662	0.4061	−0.0004331 to 0.001049
EF	0.3162	0.7648	−0.0001408 to 0.0001902
SV	0.9952	0.7649	−0.002045 to 0.002761
SVi	0.9958	0.9814	−0.002716 to 0.002654
LAVi	0.5956	0.689	−0.0003668 to 0.0005497
E/e’	0.5532	0.9798	−0.001275 to 0.001307
MW GWE	0.9964	0.3131	−0.6412 to 1.953
MW GWI	0.5085	0.5776	−7.591e-006 to 4.289e-006
MW GCW	0.9835	0.4092	−3.875e-005 to 1.610e-005
MW GWW	0.9966	0.3608	−0.0002743 to 0.0007372
Strain GLS	0.2717	**<0.0001**	−0.02044 to −0.01914
cfPWV	0.6769	**<0.0001**	0.04513 to 0.04995
ABI	0.4654	0.9791	−0.01688 to 0.01644
Ea (SBP × 0.9 / SV)	0.995	0.8105	−0.06222 to 0.07912

HT 12mo, hypertensives at 12 months; SBP, systolic blood pressure; DBP, diastolic blood pressure; MAP, mean arterial pressure; HR, heart rate; BSA, body surface area; IVST, interventricular septum thickness; PWT, posterior wall thickness; RWT, ratio of wall thickness; LVEDD, left ventricular end diastolic diameter; LVESD, left ventricular end systolic diameter; EF, ejection fraction; SV, stroke volume; SVI, stroke volume index; LAVi, left atrial volume index; MW, Myocardial Work; GWI, global work index; GCW, global constructive work; GWW, global wasted work; GWE, global work efficiency; GLS, global longitudinal strain; cfPWV, carotid femoral Pulse Wave Velocity; ABI, ankle-brachial index; Ea, arterial elastance; VAC, ventricular-arterial coupling; CI, confidence interval.

**Table 6:** MW in all its components

		Total	Male	Female
GWI (mmHg%)	Amount of myocardial work performed by the left ventricle during systole => area of PSL from mitral valve closure to mitral valve opening	1292–2505	1270–2428	1310–2538
GCV (mmHg%)	Positive work performed in systole (Shortening) + Negative work performed in isovolumetric relaxation (lengthening)	1582–2881	1650–2807	1543–2924
GWW (mmHg%)	Negative work performed in systole (lengthening) + Positive work performed in isovolumetric relaxation (shortening)	226 ± 28^a^	238 ± 33^a^	239 ±39^a^
GWE (%)	Percentage (0–100%) of constructive work over total work => Constructive work/(constructive work + wasted work)	91 ± 0.8^b^	90 ± 1.6^b^	91 ± 1^b^

Data are expressed as 95% confidence interval or limits of normality ± Standard error^a,b^.

a Highest expected value.

b Lowest expected value.

GCW = global constructive work; GWE = global work efficiency; GWI = global work index; GWW = global wasted work, PSL = pressure-strain loop.

## Summary table

### What is known about the topic

- The coupling between the heart and arteries, ventricular-arterial coupling (VAC), is a measure of overall cardiovascular performance and efficiency.
- Changes in VAC can indicate underlying heart or vascular problems.
- However, there is a lack of studies on VAC in young hypertensives without cardiovascular risk factors.
- No study has demonstrated the relevant impact of lifestyle changes and pharmacological treatment on VAC in young hypertensives without organ damage.

### What this study adds

- This study investigated VAC in young (30–49 year-old) hypertensives without organ damage vs age-matched healthy people, by assessing pulse wave velocity (PWV) and global longitudinal strain (GLS); in addition arterial elastance (Ea) and mechanical work (MW) were also investigated.
- At an early stage of hypertension three mechano-energetic components of MW, global work index (GWI), global constructive work (GCW), and global wasted work (GWW) were increased, with no signs of overt cardiovascular disease.
- VAC, Ea, PWV, and GLS did not differ between the two groups indicating no overt dysfunction in arterial stiffness or myocardial contraction.
- However, lifestyle changes and pharmacological treatment improved all the aforementioned parameters, giving additional support for early intervention in hypertension.

## Abbreviations

ABI: Ankle Brachial Index
AVC: Aortic Valve Closure
AVO: Aortic Valve Opening
BP: Blood Pressure
BSA: Body Surface Area
CBC: Complete Blood Count
cfPWV: carotid-femoral Pulse Wave Velocity
CW: Continuous Wave
DBP: Diastolic Blood Pressure
E/e’: E wave/e’ wave ratio
Ea: Arterial Elastance
EF: Ejection Fraction
ESC: European Society of Cardiology
ET: Ejection Time
GCW: Global Constructed Work
GFR: Glomerular Filtration Rate
GLS: Global Longitudinal Strain
GWE: Global Work Efficiency
GWI: Global Work Index
GWW: Global Wasted Work
Hb: Hemoglobin
HDL: High-density lipoproteins
HR: Heart Rate
HTN: Hypertensives
IMT: Intima-Media Thickness
IVC: Isovolumetric Contraction
IVR: Isovolumetric Relaxation
IVST: Interventricular Septum Thickness
LAV: Left atrial volume
LAVi: Left Atrial Volume Index
LDL: Low-density lipoproteins
LVEDD: Left Ventricular End Diastolic Diameter
LVEDV: Left Ventricular End Diastolic Volume
LVESD: Left Ventricular End Systolic Diameter
LVESV: Left Ventricular End Systolic Volume
LVOT: Left Ventricular Outflow Tract
LV: Left Ventricular
MAP: Mean Arterial Pressure
MW: Myocardial Work
MWI: Myocardial Work Index
MVC: Mitral Valve Closure
MVO: Mitral Valve Opening
Na2EDTA: disodium ethylenediaminetetraacetate
NT: Normotensives
PLT: Platelets
PSL: Pressure Strain Loop
PTT: Pulse Transit Time
PWT: Posterior Wall Thickness
PW: Pulsed Wave
PWV: Pulse Wave Velocity
ROI: Region of Interest
RPM: Revolutions per Minute
RWT: Relative Wall Thickness
SBP: Systolic Blood Pressure
SV: Stroke Volume
Svi: Stroke Volume Index
VAC: Ventricular-Arterial Coupling
WBC: White Blood Cells
